# Unraveling the impact of genetic variants

**DOI:** 10.7554/eLife.107459

**Published:** 2025-06-09

**Authors:** Bruce Herring

**Affiliations:** 1 Department of Biological Sciences, University of Southern California Los Angeles United States

**Keywords:** TRIO, neurodevelopment disorders, Rac1, presynaptic, RhoA, Mouse

## Abstract

Experiments in mice reveal how three rare mutations in a gene called *TRIO* can lead to different neurodevelopmental outcomes.

**Related research article** Ishchenko Y, Jeng AT, Feng S, Nottoli T, Manriquez-Rodriguez C, Nguyen KK, Carrizales MG, Vitarelli MJ, Corcoran EE, Greer CA, Myers SA, Koleske AJ. 2025. Heterozygosity for neurodevelopmental disorder-associated *TRIO* variants yields distinct deficits in behavior, neuronal development, and synaptic transmission in mice. *eLife* **13**:RP103620. doi: 10.7554/eLife.103620.

Neurodevelopmental disorders like autism spectrum disorder, schizophrenia, and bipolar disorder are deeply complex conditions, with each involving a mosaic of genetic and environmental contributors. A number of risk genes have been identified, but pinpointing how a particular mutation in one of these genes translates into specific neuronal and behavioral phenotypes remains a challenge.

One risk gene, known as *TRIO*, encodes a large signaling protein that contains two guanine nucleotide exchange factor (GEF) domains. One of these domains activates an enzyme called Rac1, while the other activates an enzyme called RhoA (Figure 1). These enzymes have important roles in brain development as they are involved in regulating the development of neurons, dendrites and synapses (Paskus et al., 2020).

**Figure 1.**
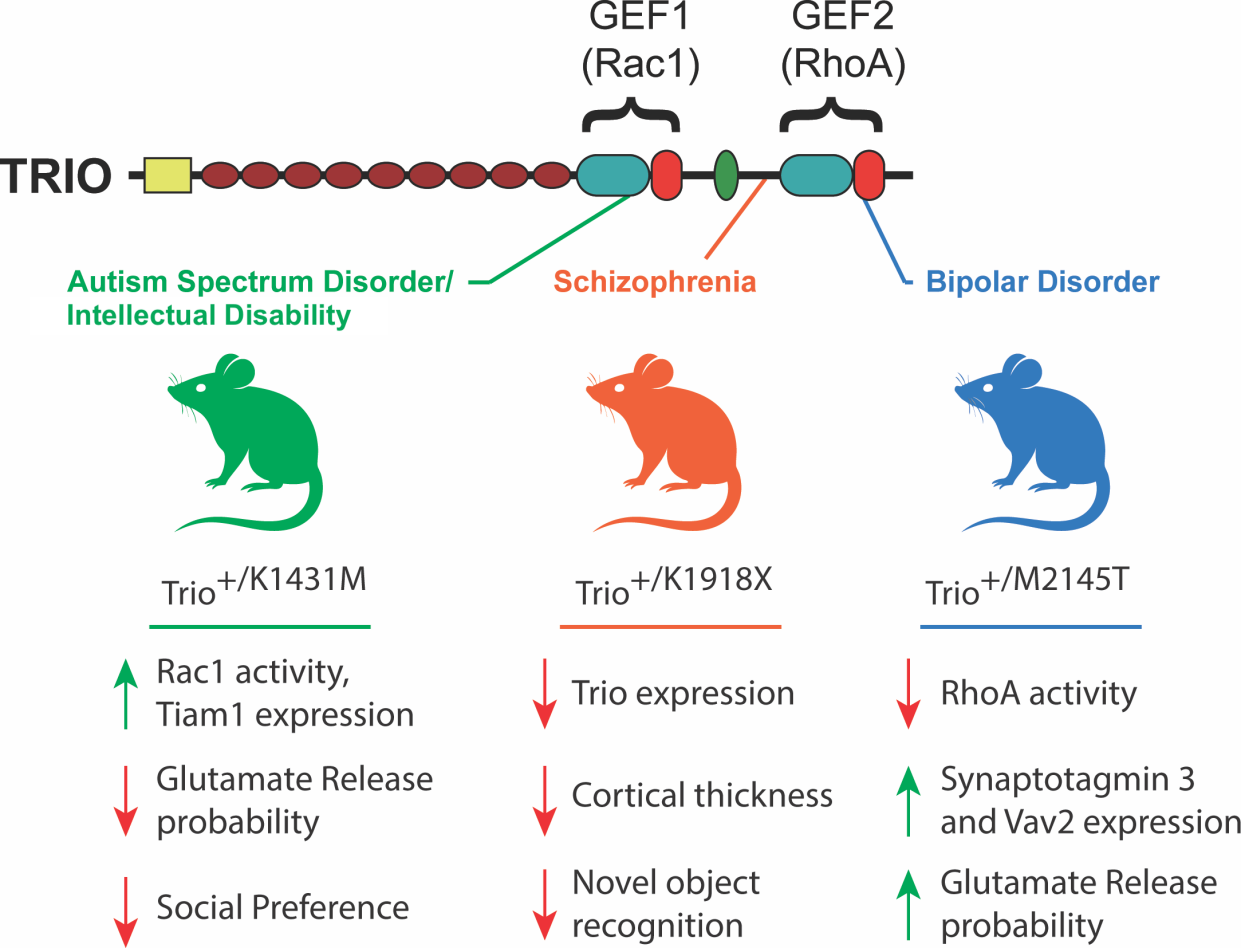
Phenotypes observed in mouse models of *TRIO* dysfunction. The GEF1 and GEF2 domains of the TRIO protein (top) activate enzymes called Rac 1 and RhoA, respectively. Ishchenko et al. generated three mouse strains (middle), each with a distinct mutation in the *Trio* gene that was associated with a specific neurodevelopmental/psychiatric disorder. The mutations were introduced in only one copy of the gene in order to mimic the genetic structure of humans with these *TRIO*-associated brain disorders. The K1431M mutation (green; left), which is associated with autism spectrum disorder, increased the activity and expression of certain proteins, while reducing the probability of glutamate release, and also reducing interactions with other mice. The K1918X mutation (orange; center), which is associated with schizophrenia, had an adverse impact on the expression of Trio, cortical thickness and memory-associated tasks. The M2145T mutation (blue; right), which is associated with bipolar disorder, reduced the activity of some proteins while increasing the expression of others, and also reduced the probability of glutamate release. GEF: guanine nucleotide exchange factor.

Importantly, different mutations in *TRIO* have been strongly linked to distinct neurodevelopmental disorders (Paskus et al., 2020; Singh et al., 2022; Genovese et al., 2016; Katrancha et al., 2017; Sadybekov et al., 2017; Barbosa et al., 2020). This makes *Trio* an exceptional model for studying how precise molecular alterations can drive varied disease phenotypes. Now, in eLife, Anthony Koleske and colleagues from Yale University, the La Jolla Institute for Immunology and the Chinese PLA General Hospital – including Yevheniia Ishchenko and Amanda Jeng as joint first authors – report the distinct effects of three different *Trio* mutations on brain development and behavior in mice (Ishchenko et al., 2025).

Ishchenko et al. began by engineering mice to carry one of three rare *TRIO* mutations: K1431M (autism spectrum disorder), K1918X (schizophrenia), and M2145T (bipolar disorder). Each mutation was present in a single copy of the gene to reflect the heterozygous nature of the human genetics, where one mutated copy is paired with a normal copy of the gene. The team then conducted a comprehensive battery of behavioral, anatomical, electrophysiological, and biochemical analyses.

The analyses revealed that each variant produced unique phenotypic profiles in mice (Figure 1). K1431M mice showed a reduced probability of releasing the neurotransmitter glutamate (indicating impaired synapse function) and robust social deficits, such as decreased time spent interacting with other mice. K1918X mutants exhibited a thinner brain cortex, reduced dendrite complexity, and impaired ability to tell novel objects from familiar objects, suggesting an impact on specific types of memory. In M2145T mice, while behavioral impacts were more subtle, there were clear synaptic transmission defects including increased glutamate release probability. Together, these findings underscore how individual mutations in the same gene can selectively perturb different aspects of brain function.

One particularly intriguing discovery came from the K1431M variant. Although this mutation reduces the ability of the Trio protein to activate Rac1, mutant mice instead showed elevated Rac1 activity in the cortex. This might be explained by increased expression of Tiam1, another protein that can activate Rac1, suggesting a compensatory response that may itself contribute to dysfunction. Pharmacological inhibition of Rac1 partially restored synaptic function in these mice, hinting at possible therapeutic avenues.

Unlike some previous studies that relied on fully knocking out or overexpressing genes, the work of Ishchenko et al. models the subtler heterozygous mutations found in individuals. The findings demonstrate that such single-copy changes are sufficient to disrupt neuronal development and behavior in mutation-specific ways.

Future studies using these rodent models should examine how such mutations alter neuronal activity patterns in mice, and how different environmental factors impact their effects. Complementary modeling in human-derived neurons may also help identify the changes that are translationally relevant, and could reveal whether other components of the Trio signaling pathway could serve as biomarkers of neurodevelopmental disorders or therapeutic targets.

More broadly, this study emphasizes the power of variant-specific modeling to decode the molecular underpinnings of neurodevelopmental and psychiatric disease. As genetic sequencing becomes increasingly integrated into clinical care, understanding the functional consequences of individual variants will be critical for translating genomic information into actionable insight.
